# Insights into *Candida* Colonization in Intensive Care Unit Patients: A Prospective Multicenter Study

**DOI:** 10.3390/jof10060378

**Published:** 2024-05-25

**Authors:** Teresa Nascimento, João Inácio, Daniela Guerreiro, Patrícia Patrício, Luís Proença, Cristina Toscano, Priscila Diaz, Helena Barroso

**Affiliations:** 1 Unidade de Microbiologia Médica, Global Health and Tropical Medicine (GHTM), Instituto de Higiene e Medicina Tropical, Universidade Nova de Lisboa, 1349-008 Lisboa, Portugal; 2 Egas Moniz Center for Interdisciplinary Research (CiiEM), Egas Moniz School of Health & Science, Caparica, 2829-511 Almada, Portugal; dguerreiro@egasmoniz.edu.pt (D.G.); lproenca@egasmoniz.edu.pt (L.P.); mhbarroso@egasmoniz.edu.pt (H.B.); 3 School of Applied Sciences, University of Brighton, Brighton BN2 4GJ, UK; j.inacio@brighton.ac.uk; 4 Hospital Beatriz Ângelo, 2674-514 Loures, Portugal; patricia.silva@hbeatrizangelo.pt; 5 Centro Hospitalar Lisboa Ocidental Hospital Egas Moniz, 1349-019 Lisboa, Portugal; ctoscano@chlo.min-saude.pt; 6 Hospital Prof. Doutor Fernando da Fonseca, 2720-276 Amadora, Portugal; priscila.diaz@hff.min-saude.pt

**Keywords:** *Candida* spp., intensive care unit, colonization, surveillance, risk factors, prevalence

## Abstract

The skin mycobiota plays a significant role in infection risk, pathogen transmission, and personalized medicine approaches in intensive care settings. This prospective multicenter study aimed to enhance our understanding of intensive care units’ (ICUs’) *Candida* colonization dynamics, identify modifiable risk factors, and assess their impact on survival risk. Specimens were taken from 675, 203, and 110 patients at the admission (D1), 5th (D5), and 8th (D8) days of ICU stay, respectively. The patient’s demographic and clinical data were collected. *Candida* isolates were identified by conventional culture-based microbiology combined with molecular approaches. Overall, colonization was 184/675 (27.3%), 87/203 (42.8%), and 58/110 (52.7%) on D1, D5, and D8, respectively. *Candida* colonization dynamics were significantly associated with ICU type (odds ratio (OR) = 2.03, 95% CI 1.22–3.39, *p* = 0.007), respiratory infection (OR = 1.74, 95% CI 1.17–2.58, *p* = 0.006), hemodialysis (OR = 2.19, 95% CI 1.17–4.10, *p* = 0.014), COVID-19 (OR = 0.37, 95% CI 0.14–0.99, *p* = 0.048), and with a poor 3-month outcome (*p* = 0.008). Skin *Candida* spp. colonization can be an early warning tool to generate valuable insights into the epidemiology, risk factors, and survival rates of critically ill patients, and should be considered for epidemiological surveillance.

## 1. Introduction

A dramatic global increase in the incidence of fungal diseases has been documented in the past 20 years, which represents a public health problem with unique challenges due to the lack of sensitivity of diagnostic tools and the high morbidity and mortality caused by these infections [1,2]. *Candida* spp. are the third leading cause of nosocomial bloodstream infections, but rank first in terms of mortality [3]. *Candida* spp. are commensal yeasts that are part of the normal human skin and gut microbiota, and they are detectable in up to 60% of healthy individuals [4]. Invasive disease is usually a consequence of increased or abnormal colonization together with a local or generalized defect in host defenses [4,5,6].

ICU patients often have multiple risk factors for invasive candidiasis (IC), including prolonged hospitalization, use of broad-spectrum antibiotics, invasive procedures, and immunosuppression [7]. The diagnosis of IC can be challenging due to several factors, including the lack of pathognomonic symptoms, and the low sensitivity of diagnostic tests, displaying a variety of prediction rules for invasive *Candida* infection [8]. *Candida* multifocal colonization has been suggested as an independent risk factor for IC and helps to distinguish between high- and low-risk patients [9]. As a result, *Candida* colonization screening based on multiple body sites has been performed routinely in many ICUs, but the body sites surveyed vary considerably [10]. The skin microbiota of patients and healthcare workers can serve as reservoirs for potential pathogens, facilitating their transmission within the healthcare setting, namely *C. parapsilosis* and *C. auris* [11].

Although *C. albicans* is still considered the most common cause of colonization/infection, over the past decade, a change in favor of non-*albicans Candida* (NAC) has been confirmed in many studies worldwide, with regional heterogeneity [12]. In addition, the rise of fluconazole-resistant non-*albicans Candida* species, such as *Candida auris* [13], has caused great concern for healthcare across the globe, making the establishment of adequate empiric antifungal regimens difficult [14].

This study aimed to understand the dynamics of skin mycobiota colonization among ICU patients and evaluate the association between *Candida* colonization and the patient’s outcome.

## 2. Materials and Methods

### 2.1. Study Population

This study was conducted in three ICUs of two tertiary care hospitals in the metropolitan Lisbon area, Portugal. *Hospital Professor Doutor Fernando Fonseca* (FFH) is an 802-bed hospital with two ICUs (general and surgery) and *Beatriz Ângelo Hospital* (BAH) is a 424-bed hospital with one general ICU. Patients admitted to the ICU between January 2020 and December 2022 were enrolled in the study with the following exceptions: patients under 18 years of age, pregnant women, and mentally disabled individuals. The patient cohort met at least one of the following inclusion criteria: antimicrobial therapy in the last 48 h, presence of a central intravascular catheter, receiving parenteral nutrition, undergoing hemodialysis, receiving invasive assisted ventilation, having a bladder catheter, recent surgery, diagnosis of HIV/AIDS, other forms of immunodeficiency, hematological malignancies, other types of cancer, neutropenia (<500/mm^3^), anemia with hemoglobin levels below 10 mg/dL, and diabetes.

The study was approved by both Hospitals Hospital Ethics Committee. The sampling of each patient was performed by a non-invasive, bilateral axillary/inguinal combined swab. Collections were made upon the admission of patients to the ICU (D1) and continued during the ICU stay: 5th day (D5) and 8th day (D8), when applicable.

The sequential timing of collecting samples (D1, D5, and D8) during the ICU stay was undertaken with the objective of closely observing the influence of the ICU setting, providing insights into the progression of colonization and the effectiveness of intervention strategies [15,16].

### 2.2. Data Collected for Analysis

Demographic data, such as age, gender, history of travel abroad, underlying diseases [pulmonary infection, cardiovascular disease, gastrointestinal pathology, urinary tract infection, solid tumor, HIV/AIDS, hematological malignancy (lymphoma, leukemia, and another neoplasm), severe immunodeficiency, neutropenia, hemoglobin <10 g/dL, diabetes, and COVID-19], the history of antibiotic and antifungal therapy, and individual host risk factors related to the ICU setting [central intravascular catheter (CVC), invasive assisted ventilation, abdominal surgery, hemodialysis (HD), total parenteral nutrition (TPN), and vesical catheter] were recorded.

### 2.3. Surveillance Cultures and Identification

Swabs were collected in liquid Amies transport medium and 50 µL aliquots of the suspensions were spread directly onto appropriate culture media: Sabouraud Gentamicin Chloramphenicol 2 agar (SDA) (bioMérieux, Marcy l’Etoile, France) and a commercially *Candida* Chromogenic Medium (CHROMagar ^TM^ *Candida*, CHROMagar, Paris, France). Plates were incubated aerobically for 48 h, one set of plates at 25 °C and a second set at 37 °C, and the colony-forming units (CFUs) counted.

**Figure 1:**
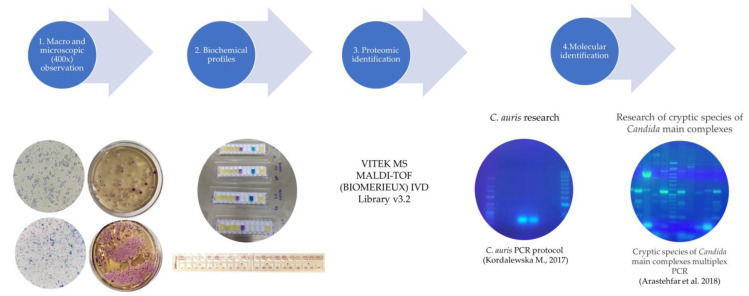
Workflow of the identification procedures used with *Candida* spp. isolates [18,19].

The presumptive identification of isolates was based on standard criteria of macroscopic and microscopic morphologies, growth temperature, biochemical profile of aerobic sugar assimilation, and appearance on chromogenic agar (Figure 1). All isolates were further processed for analysis with MALDI-TOF MS—VITEK MS (bioMérieux, Marcy l’Etoile, France) using VITEK MS v3.2 software [17]. All identifications displaying a single result with a confidence value of 99.9% were considered acceptable (Figure 1).

All *Candida* isolates were also subjected to a *C. auris*-specific polymerase chain reaction (PCR) assay [18] and screened to identify any potential *Candida* cryptic species from the main complexes [19]. For this purpose, total DNA was extracted from the isolates using a NZYMicrobial gDNA Isolation Kit^®^ (Nzytech, Lisboa, Portugal), according to the manufacturer’s instructions. Primers used in both PCR assays were previously described [15,16] and synthesized by Stab Vida, Portugal. PCRs were performed in a T100 thermal cycler (Bio-Rad Laboratories, Inc., Hercules, CA, USA). Amplified products were analyzed using 2% agarose gels stained with greensafe (Nzytech, Portugal) and visualized on a UV transilluminator with an exposure time of 4 s (Figure 1).

### 2.4. Quantification of Growth

Isolates recovered from swab samples were subjected to quantification based on the initial volume of sample spread. Counts were delineated to correspond with visual thresholds, ensuring practicality and accuracy in the assessment process. Colonization density was distributed into three groups: <100 CFU/mL; 100–1000 CFU/mL; and >1000 CFU/mL [20]. Namely, counts below 100 CFU/mL were categorized as a maximum of 5 CFUs, those between 100 and 1000 CFU/mL (maximum of 50 CFUs), and counts exceeding 1000 CFU/mL (>50 CFUs). High colonization was defined by the detection of more than 50 CFUs, as previously described [21]. Cultures were visually examined at 24 h and 48 h and an evaluation was performed independently by two different qualified investigators.

### 2.5. Statistical Analysis

A database was created with the demographic, clinical, and mycological characteristics of the study group, and data were analyzed using the IBM SPSS Statistics v. 29.0 (IBM Corp., Armonk, NY, USA) package program. An exploratory and descriptive analysis of the data was carried out to identify patterns for each variable. The categorical variables were expressed as frequencies and percentages. Comparisons of categorical variables were performed using the chi-squared test. Univariable and multivariable logistic regression models were performed to identify the predictors of *Candida* colonization at admission and during the whole length of the ICU stay. A *p*-value < 0.05 was taken to be statistically significant for all the above inferential analyses.

## 3. Results

### 3.1. Demographic and Clinical Characteristics of Patients

A total of 675 patients were enrolled during the two-year study period, 2020–2022, with 71, 64, and 540 patients attending, respectively, the general FFH ICU, surgical FFH ICU, and BAH ICU. From this cohort, all patients who met the inclusion criteria upon admission and remained hospitalized throughout the study period were followed up. A total of 203 patients were followed up with a second collection on day 5 (D5), and, from these, 89 patients were further sampled at day 8 (D8). Twenty-one patients were sampled at D1 and D8 (but not D5). Overall, 988 swab samples were collected: 675 on admission, 203 on D5, and 110 on D8.

A total of 401 patients (60.0%) were male and 566 (83.0%) were leucodermic with a median age of 67 years. A significant percentage had relevant underlying comorbidities at admission to each ICU, namely pulmonary infection for general FFH ICU; solid organ tumors and hematological neoplasms for surgical FFH ICU; and cardiovascular pathology, anemia, immunodeficiency, and COVID-19 for BAH ICU. Risks factors associated with the ICU setting are relevant for surgical the FFH ICU and include the presence of a central venous catheter, mechanical ventilation, and abdominal surgery. The use of antibiotics and antifungals can also be seen to be significant for the FFH ICU in this cohort. Antifungal use was for prophylaxis (fluconazole or echinocandins) and considered only in targeted patient groups, namely, patients with recent abdominal surgery and recurrent gastrointestinal perforations or diabetic (Table S1). The complete demographic and clinical characteristics of the study population is reported in Table 1.

### 3.2. Rates of Colonization

Fungal species were isolated from 184/675 (27.3%), 87/203 (42.9%), and 58/110 (52.7%) patients, respectively upon admission, D5 and D8. Increased colonization was observed in patients monitored during the whole length of stay.

The dynamics of colonization expressed in terms of variations with time of stay in ICU evidenced that, from the 203 patients with collection at D5, 74 (36.5%) were previously colonized at D1. For the 110 patients with collection at D8, 48/110 (43.6%) and 42/110 (38.2%) were already colonized, respectively, at D1 and D5 (Table 2). The overall colonization showed 232/675 (34.4%) colonized patients, whereby 48/313 (15.3%) became colonized in the ICU and 26/89 (29.2%) stayed colonized during the whole length of stay at the ICU (Table 2).

### 3.3. Burden of Colonization

**Figure 2:**
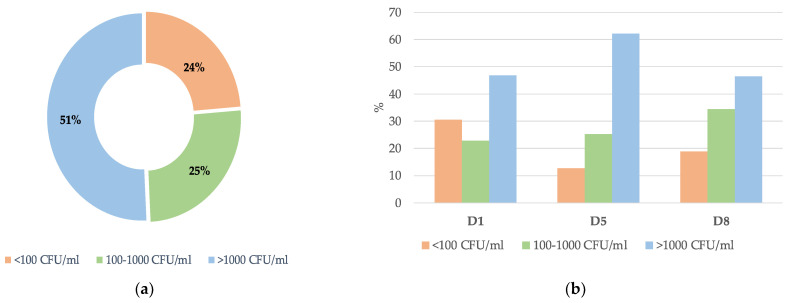
Distribution of colonization density in the ICU: (**a**) overall; (**b**) on admission day (D1); day 5 (D5); and day 8 (D8).

From the 329 positive samples for fungi isolation, 167 (50.8%), 84 (25.4%), and 78 (23.8%) presented, respectively, with a high fungal density (>1000 CFU/mL), average density (100–1000 CFU/mL), and low density (100 CFU/mL) (Figure 2a).

The rate of colonization throughout the ICU stay reflected some variations depending on the level of CFU/mL. For patients with a rate of colonization <100 CFU/mL, there was a slight decrease after admission to the ICU and an increase after D5 of hospitalization (D1: 30.4%; D5: 12.6%; and D8: 19.0%). For intermediate colonization values in the range of 100–1000 CFU/mL, there was an increase along the prevalence points (D1: 22.8%; D5: 25.3%; and D8: 34.5%). The densely colonized samples (>1000 CFU/mL) gradually increased until D5 of hospitalization and by D8 showed similar colonization rates to D1 (D1: 46.7%; D5: 62.1%; and D8: 46.6%) (Figure 2b). However, no statistical differences were found at each of the collection points and the level of CFU/mL (D1, *p* = 0.223; D5, *p* = 0.939; and D8, *p* = 0.669).

### 3.4. Diversity of Colonizing Species

A total of 371 isolates were obtained from the 329 culture-positive samples. Most samples yielded single isolates, 286/329 (86.9%), and 43/329 (13.1%) samples yielded mixed cultures with two or more fungal species present. Mixed cultures were particularly observed at D1, with a decrease over the collection points [D1 (25/43, 58.1%); D5 (12/43, 27.9%); and D8 (6/43, 14.0%)].

Four genera of yeast-like fungi were found with a predominance of *Candida* spp., 355/371 (95.7%), followed by *Rhodotorula* spp., 9/371 (2.4%), *Trichosporon* spp., 6/371 (1.6%), and *Saccharomyces* spp., 1/371 (0.3%).

**Figure 3:**
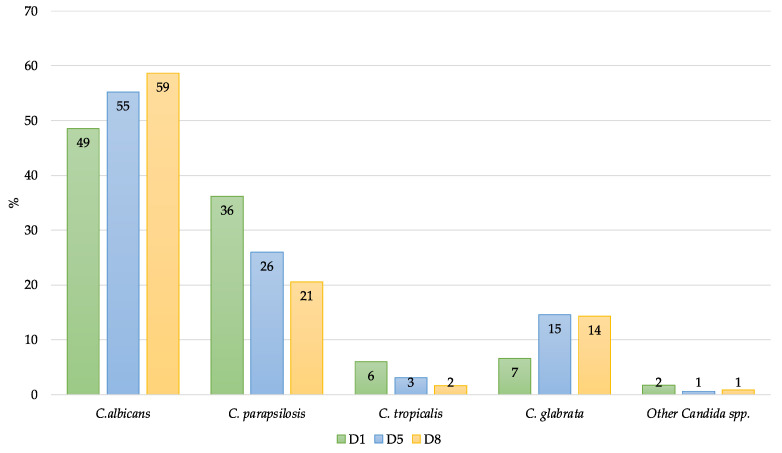
Diversity of *Candida* spp. during the whole length of ICU stay (D1, D5, and D8).

After identification to the species level, eight *Candida* species were identified: *C. albicans* sensu stricto (*n* = 185), *C. parapsilosis* complex (*n* = 112) [*C. parapsilosis* sensu stricto (*n* = 109), *C. orthopsilosis* (*n* = 2), *C. metapsilosis* (*n* = 1)], *Nakaseomyces glabrata* (*Candida glabrata)* sensu stricto (*n* = 36), *C. tropicalis* (*n* = 15), *Clavispora lusitaniae* (*Candida lusitaniae*) (*n* = 4), and *Meyerozyma guilliermondii* (*Candida guilliermondii*) (*n* = 3). *C. auris* or other emerging *Candida* species, like the *C. haemulonii* complex, *C. rugosa*, or *C. vulturna*, were not detected. *C. albicans* remained the most isolated species with 185/355 (52.1%). The distribution of *Candida* spp. Over the collection points evidenced a relevant colonization by *C. albicans* in the ICU setting (D5 and D8) at the expense of non-*albicans* species (Figure 3).

### 3.5. Demographics, Clinical Characteristics, and Outcomes of Patients Colonized

We observed that gender, race, and patient age did not pose a positive impact on the fungal colonization density rates. As summarized in Table 3, at ICU admission, the most susceptible patients to colonization were admitted to FFH general ICU, accounting for 29/71 (40.8%) (*p* = 0.007). Importantly, among the various relevant risk factors, which significantly influenced the incidence of *Candida* colonization and possible infection, pulmonary infection, the presence of CVC, mechanical ventilation, and dialysis were found to be statistically significant (*p* < 0.05) (Table 3). Other risk factors did not show significant differences (*p* > 0.05), including urinary catheterization, abdominal surgery, total parenteral nutrition, neutropenia, cancer (leukemia and solid tumor), anemia, diabetes mellitus, and treatment with antibiotics and antifungals (Table 3).

Considering the univariate risk evaluation for permanent colonization during the whole length of stay at the ICU (D1–D8), no significant association was observed for colonization in both tertiary hospitals, including the ICU unit (Table S2).

Multivariate analysis was performed to detect potential risk factors associated with fungal colonization. Subjacent pulmonary infection and being on dialysis had statistical significance as increased risk predictors toward *Candida* colonization (OR = 1.74, CI 95% 1.17–2.58, *p* = 0.006 and OR = 2.19, 95% CI 1.17–4.10, *p* = 0.014, respectively), as did the presence of COVID-19 (OD = 0.37, 95% CI 0.14–0.99, *p* = 0.048), but in this case, lowering the risk toward colonization (Table 4).

Regarding patient outcomes, the three-month survival rate was determined at the BAH ICU for a one-year collection period (n = 497), between September 2021 and September 2022. The fatality rate was 161/497 (32.4%). No statistically significant associations were identified between risk factors for colonization already identified in this study or other risk factors related to underlying conditions or the ICU. Fatality was significantly higher in patients colonized at ICU admission (*p* = 0.010) and with a longer length of stay in the ICU (*p* = 0.006). The results show that being colonized with *C. albicans* is associated with a poor outcome (*p* = 0.042). However, the analysis did not reveal a significant association between colonization density and survival rates (*p* = 0.132) (Table 5).

From the one-year BAH cohort, it is worth mentioning that 17 patients were colonized during the whole length of stay, and 8/17 (47.0%) had a poor 3-month outcome.

## 4. Discussion

There has been a significant increase in the incidence of invasive fungal infections worldwide, especially in patients admitted to intensive care units [3]. Among different fungal pathogens, colonization by *Candida* species is very frequent in ICU patients and a necessary first step in the pathogenesis of systemic infection.

Collecting samples upon admission provided a baseline understanding of the initial *Candida* colonization status of patients entering the ICU. This helped establish a starting point for comparison with subsequent samples [16]. The one-week mark after ICU admission (D5 and D8) allowed us to access the effectiveness of interventions implemented during the ICU stay on *Candida* colonization.

The prevalence values of *Candida* colonization were 27.3%, 42.9%, and 52.7%, respectively, upon admission, D5 and D8. During the whole length of ICU stay, 15.3% more of the patients evidenced newly acquired fungal colonization. Out of the 89 patients who underwent sampling at the three specified time points, 29.2% were consistently positive for fungi, which demonstrates the importance of the ICU environment in *Candida* colonization.

Our results are consistent with a progressive increase in *Candida* colonization in the ICUs. Although colonization on admission may reflect the previous colonization of patients, it increased during the study period. Prevalence reported by other studies over the last decade point toward increasing *Candida* prevalence, but these studies observed different rates of colonization. Studies by Ahmad et al., Charles et al., and León et al. showed higher proportions of patients, respectively, 45.6%, 39.1%, and 52.2%, already colonized at the time of admission to the ICU [22,23,24]. The overall prevalence in our study (232/675, 34.4%) is consistent with a previous observational study in a surgical and trauma ICU in a university hospital [20]. However, comparing colonization rates with previous studies presents a challenge because those studies focused on determining *Candida* colonization rates in different types of septic samples just on ICU admission.

To the authors’ knowledge, this is the first Portuguese multicenter observational and descriptive study that provides insights into *Candida* colonization and predictive risk factors in an ICU setting. Previous studies conducted in Portugal have predominantly focused on cases of candidemia, often being retrospective in nature [25,26,27]. Nevertheless, comparing the overall distribution of *Candida* species with previous Portuguese candidemia studies, a steady pattern of *Candida* species ranks yielded by Portuguese ICU patients was observed [28]. Namely, a prevalence of *C. albicans* alongside the occurrence of cryptic species only from the *C. parapsilosis* complex [26,28,29].

Colonization densities were found to be mostly high in our study (>1000 CFU/mL) (167/329; 50.8%), but did not change significantly over the first 8 days of stay in intensive care unit patients, as already described by other authors [22]. This might be due to a short time of surveillance, as other studies evidenced that patients colonized with *Candida* had a significantly longer length of ICU and hospital stays [30].

Our results suggest that the risk of being colonized is dependent on the healthcare unit. The probability of a patient being colonized in the general FFH ICU is significant, compared to the other ICUs under study (*p* = 0.004). It is noteworthy that the assessed patient populations are very heterogeneous with a significant association within clinical characteristics between ICUs. Regarding the colonization results that reflect the colonization acquired in each ICU (collections on D5 and D8), the rate did not differ significantly between the ICUs. These rates for *Candida* colonization and respective density are suggestive of association with increasing exposure to risk factors and the local unit.

*Candida* colonization and infection are almost indistinguishable in the natural history of candidemia, and multiple-site colonization by *Candida* species is commonly recognized as a major risk factor for invasive fungal infection in critically ill patients [12,16]. Several risk factors have been associated with *Candida* colonization, namely, extremes of age (low-birthweight newborns and the elderly), hematological neoplasms and other cancers, chemotherapy, neutropenia, digestive tract mucositis, intravenous catheters and/or long-term treatment with corticosteroids, and even antimicrobials, including antifungal drugs [31].

For our cohort of patients, logistic regression analysis revealed significant independent predictors of *Candida* colonization in the ICU and identified three risk factors mostly related to medical interventions during intensive care or to comorbid conditions, namely an underlying respiratory infection and being under hemodialysis. These results agree with previous studies, considering respiratory disease as one of the best risk predictors for *Candida* infection development [7,32,33].

Patients with coronavirus disease 2019 (COVID-19) were also significantly associated but, contrary to our expectations, COVID-19 had a protective effect against the development of *Candida* colonization. COVID-19 patients in the ICU receive intensive medical care and these practices can help prevent *Candida* colonization from the skin mycobiome or the ICU environment. It is described that COVID-19 patients present multiple reasons to be at a higher risk for developing candidemia due to the need for intensive care management [34,35]. Our results, showing different but interesting effects of COVID-19 on critically ill patients, should be confirmed by further studies.

It is noteworthy that, for pointed risk factors, all ORs were small, which is in line with a recent meta-analysis that identified 29 risk factors for invasive *Candida* infection in ICUs from 34 studies, with most ORs small [7].

No statistically significant association was found in our study for risk factors predicting *Candida* colonization for all lengths of stay in the ICU. The results obtained are not in line with previously published studies, since there was no statistically significant association between *Candida* colonization and the presence of risk factors, such as diabetes, abdominal surgery, HIV, hematological neoplasms, solid tumors, parental nutrition, abdominal surgery, and use of extended-spectrum antibiotics [7,35,36]. This discrepancy may be due to the biological sample used in this research, combined axilla/groin swabs, while other studies used blood samples.

Our findings for 3-month mortality for the ICU under evaluation suggested a statistically significant association with the length of ICU stay and colonization by *C. albicans*, which is consistent with the observations of other studies that validated the incidence value of *Candida* colonization [16,33,37,38,39]. *C. albicans* is the main cause of IC worldwide, except in India [40], and over the infection process uses multiple virulence factors. Although *C. albicans* shows a good susceptibility profile to antifungal drugs, the in vivo response is greatly hampered by the presence of biofilm. Nevertheless, its persistence of colonization over time may be related to its potent infective potential [41]. However, there is a limitation in our study as we do not know if IC/candidemia was the sole reason that contributed or not to death in our patient cohort. With the clinical data provided, we do not have enough information to assume that colonization does or does not increase the possibility of developing candidemia and invasive infection, but we found a significant association between a poor prognosis and *Candida* colonization.

When the number of *Candida* CFUs present in patient samples was monitored over time to assess the extent of colonization, no significant association with a poor 3-month outcome was found. While high CFU counts may indicate increased colonization, it seems that they do not necessarily associate directly with the clinical outcome, as other factors, such as host immunity and underlying medical conditions, also play a role. Nevertheless, our results from the burden of colonization (D1 to D8) show that healthcare units should consider the implementation of infection control measures to mitigate the risks associated with *Candida* colonization.

Clinicians and healthcare systems can address the progressive increase in *Candida* colonization in ICUs by employing several key strategies. Firstly, they should prioritize a regular analysis of trends and patterns to detect emerging issues early, allowing for timely intervention. Rapid diagnostic tests should be utilized to promptly identify *Candida* colonization in ICU patients, enabling a swift initiation of appropriate management. Additionally, infection control practices must be strengthened, particularly emphasizing hand hygiene and environmental cleaning. Special attention should be paid to patients undergoing hemodialysis and those with respiratory infections, with tailored measures implemented to mitigate the risk of *Candida* colonization and transmission in these populations. By implementing these measures, clinicians and healthcare systems can effectively combat the rising prevalence of *Candida* colonization in ICUs and improve patient outcomes.

We showed that monitoring *Candida* CFU colonization burden in the ICUs, along with other relevant clinical and epidemiological data, can provide valuable insights into the risk factors and dynamics of *Candida* colonization in the hospital setting. In view of the results, it is possible to consider skin *Candida* colonization as an early warning tool in critically ill patients and can be considered the site of choice for epidemiological surveillance of critically ill patients.

## Figures and Tables

**Table 1:** Demographic and clinical characteristics of patients by hospital ICU ^1^.

Patient Characteristics	All Patients (n = 675)	General FFH ICU (n = 71)	Surgical FFH ICU (n = 64)	BAH ICU (n = 540)	*p*
Age range (years)					
18–40	73 (10.8)	13 (18.3)	5 (7.8)	55 (10.2)	0.210
41–60	168 (24.9)	20 (28.2)	15 (23.4)	133 (24.6)	
61–80	361 (53.5)	29 (40.8)	39 (60.9)	293 (54.3)	
81+	73 (10.8)	9 (12.7)	5 (7.8)	59 (10.9)	
Gender					
Male	401 (60.0)	30 (42.3)	34 (53.1)	337 (63.2)	0.002
Female	267 (40.0)	41 (57.7)	30 (46.9)	196 (36.8)	
Race					
Leucodermic	566 (83.0)	55 (77.5)	49 (76.6)	463 (85.7)	0.047
Melanodermic	109 (16.1)	16 (22.5)	15 (23.4)	77 (14.3)	
Travel abroad ^2^	13 (1.9)	2 (2.8)	3 (4.7)	8 (1.5)	0.178
Underlying comorbidities					
Pulmonary infection	149 (22.1)	39 (54.9)	22 (34.4)	88 (16.3)	<0.001
Cardiovascular disease	140 (20.7)	3 (4.2)	2 (3.1)	136 (25.2)	<0.001
Gastrointestinal pathology	107 (15.9)	2 (2.8)	2 (3.1)	38 (7.0)	0.471
Urinary tract infection	44 (6.5)	4 (5.6)	3 (4.7)	37 (6.9)	0.763
Solid tumor	132 (19.6)	5 (7.0)	18 (28.1)	109 (20.2)	0.006
Hematological neoplasms	11 (1.6)	0 (0.0)	5 (7.8)	6 (1.1)	<0.001
Diabetes *mellitus*	167 (24.7)	16 (22.5)	13 (20.3)	138 (25.6)	0.591
HIV/AIDS	13 (1.9)	0 (0.0)	2 (3.1)	11 (2.0)	0.384
Anemia (Hb <10 mg/dL)	68 (10.1)	3 (4.2)	1 (1.6)	64 (11.9)	0.008
Severe immunodeficiency	11 (1.6)	0 (0.0)	0 (0.0)	11 (2.0)	<0.001
COVID-19	35 (5.2)	0 (0.0)	0 (0.0)	35 (6.5)	0.010
Risk factors					
Presence of CVC ^3^	357 (52.9)	39 (54.9)	43 (67.2)	275 (50.9)	0.045
Mechanical ventilation	210 (31.1)	27 (38.0)	29 (45.3)	154 (28.5)	0.010
TPN ^4^	9 (1.3)	1 (1.4)	0 (0.0)	8 (1.5)	0.619
Abdominal surgery	194 (28.7)	9 (12.7)	23 (35.9)	162 (30.0)	0.004
Neutropenia	6 (0.9)	0 (0.0)	0 (0.0)	6 (1.1)	0.469
Vesical catheter	419 (62.1)	47 (66.2)	47 (73.4)	325 (60.2)	0.089
Dialysis	45 (6.7)	5 (7.0)	6 (9.4)	34 (6.3)	0.641
Antibiotic therapy ^5^	313 (46.4)	56 (78.9)	45 (70.3)	212 (39.3)	<0.001
Antifungal therapy ^6^	16 (2.4)	2 (2.8)	5 (7.8)	9 (1.7)	0.009

^1^ Data are presented as No. (%) unless otherwise specified. ^2^ In the past three months. ^3^ Central venous catheter (CVC). ^4^ Total parenteral nutrition (TPN). ^5^ In the past 48 h. ^6^ Clinical characteristics of patients under antifungal therapy (Table S1).

**Table 2:** Dynamics of patient stay at ICU vs. colonization rate, from D1 to D8 ^1^.

Patient Cohort	D1 (n = 675)	D5 (n = 203)	D8 (n = 110)
Colonized (total)	184 (27.3)	87 (42.9)	58 (52.7)
Previously colonized (at D1)	-	74 (36.5)	48 (43.6)
Previously colonized (at D5)	-	-	42 (38.2)
Previously colonized (at both D1 and D5)	-		26 (29.2) ^2^
First colonized	-	36 (17.7)	12 (10.9)

^1^ Data are presented as No. (%) unless otherwise specified. ^2^ A total of 29.2% out of 89 patients that remained during the whole length of stay at the ICU was permanently colonized.

**Table 3:** Univariate risk evaluation toward patient colonization at admission to the ICU *.

Variable	Categories	Colonized n (%)	Odds Ratio (95% CI)	*p*
ICU Unit	BAH	137/540 (25.4)	1	-
	Surgical FFH	18/64 (28.1)	1.15 (0.65–2.05)	0.634
	General FFH	29/71 (40.8)	2.03 (1.22–3.39)	0.007
Age range (years)	18–40	23/73 (31.5)	1	-
	41–60	43/168 (25.6)	0.75 (0.41–1.37)	0.345
	61–80	94/361 (26.0)	0.76 (0.44–1.32)	0.338
	81+	24/73 (32.9)	1.06 (0.53–2.13)	0.859
Gender	Female	78/267 (29.2)	1	-
	Male	105/401 (26.2)	0.86 (0.61–1.21)	0.390
Race	Melanodermic	26/107 (24.3)	1	1
	Leucodermic	158/566 (27.9)	1.21 (0.75–195)	0.442
Diabetes mellitus	No	130/508 (25.6)	1	-
	Yes	54/167 (32.3)	1.34 (0.95–2.03)	0.090
Pulmonary infection	No	131/526 (24.9)	1	-
	Yes	53/149 (35.6)	1.67 (1.13–2.46)	0.010
Cardiovascular disease	No	151/536 (28.2)	1	-
	Yes	33/139 (23.7)	0.79 (0.51–1.22)	0.296
Solid tumor	No	147/543 (27.1)	1	-
	Yes	37/132 (28.0)	1.05 (0.69–1.60)	0.824
Gastrointestinal pathology	No	157/570 (27.5)	1	-
	Yes	27/105 (25.7)	0.91 (0.57–1.46)	0.699
Anemia (Hb < 10 mg/dL)	No	161/607 (26.5)	1	-
	Yes	23/68 (33.8)	1.42 (0.83–2.41)	0.200
Urinary tract infection	No	170/631 (26.9)	1	-
	Yes	14/44 (31.8)	1.23 (0.66–2.44)	0.482
COVID-19	No	179/640 (28.0)	1	-
	Yes	5/35 (14.3)	0.43 (0.16–1.12)	0.085
HIV/AIDS	No	183/662 (27.6)	1	-
	Yes	1/13 (7.7)	0.22 (0.03–1.69)	0.145
Hematological malignancy	No	180/664 (27.1)	1	-
	Yes	4/11 (36.4)	1.54 (0.44–5.31)	0.497
Severe immunodeficiency	No	182/664 (27.4)	1	-
	Yes	2/11 (18.2)	0.59 (0.13–2.75)	0.500
Vesical catheter	No	64/256 (25.0)	1	-
	Yes	120/419 (28.6)	1.20 (0.85–1.71)	0.303
Presence of CVC	No	74/318 (23.3)	1	-
	Yes	110/357 (30.8)	1.47 (1.04–2.07)	0.028
Mechanical ventilation	No	114/465 (24.5)	1	-
	Yes	70/210 (33.3)	1.54 (1.08–2.20)	0.018
Abdominal surgery	No	139/481 (28.9)	1	-
	Yes	45/194 (23.2)	0.74 (0.50–1.10)	0.132
Dialysis	No	165/630 (26.2)	1	-
	Yes	19/45 (42.2)	2.06 (1.11–3.82)	0.020
Total parenteral nutrition	No	181/666 (27.2)	1	-
	Yes	3/9 (33.3)	1.34 (0.33–5.41)	0.681
Neutropenia	No	182/669 (27.2)	1	-
	Yes	2/6 (33.3)	1.34 (0.24–7.37)	0.738
Antibiotic therapy	No	88/362 (24.3)	1	-
	Yes	96/313 (30.7)	1.38 (0.98–1.93)	0.065
Treatment with antifungal agents	No	179/659 (27.2)	1	-
	Yes	5/16 (31.3)	1.22 (0.42–3.56)	0.717

* A total of 184 colonized patients at D1: 27.3% from total (N = 675).

**Table 4:** Multivariate risk evaluation towards patient colonization at admission to the ICU *.

Predictor		Odds Ratio (95% CI)	*p*
Pulmonary infection	Yes	1.74 (1.17–2.58)	0.006
Dialysis	Yes	2.19 (1.17–4.10)	0.014
COVID-19	Yes	0.37 (0.14–0.99)	0.048

* Final reduced model obtained by a forward stepwise (Wald) procedure. The model was statistically significant, χ2 (3) = 16.405, *p* < 0.001, with Nagelkerke R^2^ = 0.035 and correctly classified 73.0% of cases.

**Table 5:** Survival risk evaluation (3-month outcome) for colonization during the whole length of stay at the Beatriz Ângelo Hospital (BAH) ICU (D1–D8) ^1^.

ICU Patients *n* = 497	Total	Survived n = 336	Died n = 161	*p*
Predictor				
Pulmonary infection	83/497 (16.7)	52/336 (15.5)	31/161 (19.3)	0.291
Dialysis	32/497 (6.4)	21/336 (6.3)	11/161 (6.8)	0.061
COVID-19	33/497 (6.6)	25/336 (7.4)	8/161 (5.0)	1.073
Antifungal for prophylaxis ^2^				
Fluconazole	8/497 (1.6)	4/336 (1.2)	4/161 (2.5)	0.283
Echinocandins	1/497 (0.2)	0/336 (0.0)	1/161 (0.6)	0.148
Collection day				
D1	130/497(26.2)	76/336 (22.6)	54/161 (33.5)	0.010
D5	53/129 (41.1)	34/336 (10.1)	19/161 (11.8)	0.570
D8	38/68 (55.9)	18/336 (5.4)	20/161 (12.4)	0.006
*Candida* spp.				
*C. albicans*	78/497 (15.7)	45/336 (13.4)	33/161 (20.5)	0.042
*C. parapsilosis*	48/497 (9.7)	30/336 (8.9)	18/161 (11.2)	0.426
*C. glabrata*	11/497 (2.2)	6/336 (1.8)	5/161 (3.1)	0.349
*C. tropicalis*	6/497 (1.2)	5/336 (1.5)	1/161 (0.6)	0.408
CFU/mL (>1000)	64/497 (12.9)	38/336 (11.3)	26/161 (16.1)	0.132

^1^ Data are presented as No. (%) unless otherwise specified. ^2^ Out of the nine patients, only one developed candidemia.

## Data Availability

The original contributions presented in the study are included in the article/Supplementary Materials, further inquiries can be directed to the corresponding author.

## Supplementary Materials

The following supporting information can be downloaded at: https://www.mdpi.com/article/10.3390/jof10060378/s1. Table S1: Demographic and clinical characteristics of patients under prophylactic antifungal therapy by hospital ICU. Table S2: Univariate risk evaluation for permanent colonization during the whole length of stay at the ICU (D1–D8).
