# Supplementary material for: Insights into Candida Colonization in Intensive Care Unit Patients: A Prospective Multicenter Study
Source: J Fungi (Basel). 2024 May 25;10(6):378. doi: 10.3390/jof10060378 (PMC11204936; doi:10.3390/jof10060378)
Supplement: Supplementary file 1 [file jof-10-00378-s001.zip › jof-3012750-supplementary.pdf]

Journal of Fungi

MDPI

Table S1. Demographic and clinical characteristics of patients under prophylactic antifungal therapy by hospital ICU[1].

Patient characteristics	All Patients (n=16)	General FFH ICU (n=2)	Surgical FFH ICU (n=5)	BAH ICU (n=9)	*p*
**Underlying comorbidities**					
Pulmonary infection	7 (43.8)	2 (100)	3 (60.0)	2 (22.2)	0.091
Gastrointestinal pathology	2 (12.5)	0 (0.0)	1 (20.0)	1 (11.1)	0.756
Solid tumor	6 (37.5)	1 (50.0)	3 (60.0)	2 (22.2)	0.348
Hematological neoplasms	1 (6.3)	0 (0.0)	1(20.0)	0 (0.0)	0.309
Diabetes *mellitus*	7 (43.8)	1 (50.0)	2 (40.0)	4 (44.4)	0.969
Anemia (Hb <10mg/dl)	2 (12.5)	0 (0.0)	0 (0.0)	2 (22.2)	0.411
Severe immunodeficiency	1 (6.3)	0 (0.0)	0 (0.0)	1 (11.1)	0.660
COVID-19	0 (0.0)	0 (0.0)	0 (0.0)	0 (0.0)	-
**Risk factors**	12 (75.0)	1 (50.0)	5 (100.0)	6 (66.7)	0.264
Presence of CVC[3]	6 (37.5)	0 (0.0)	2 (40.0)	4 (44.4)	0.497
Mechanical ventilation	1 (6.3)	0 (0.0)	0 (0.0)	1 (11.1)	0.660
TPN[4]	4 (25.0)	0 (0.0)	1 (20.0)	3 (33.3)	0.587
Abd. Surgery	13 (81.3)	1 (50.0)	5 (100)	7 (77.8)	0.285
Vesical catheter	2 (12.5)	0 (0.0)	1 (20.0)	1 (11.1)	0.756
Dialysis					
Antibiotic therapy[5]	14 (87.5)	2 (100.0)	5 (100.0)	7 (77.8)	0.411
Antifungal therapy					0.279
Fluconazole	11 (68.8)	1 (50.0)	2 (40.0)	8 (88.9)	
Caspofungin	4 (25.0)	1 (50.0)	2 (40.0)	1 (11.1)	
Fluc+Casp	1 (6.3)	0 (0.0)	1 (20.0)	0 (0.0)	

Table S2. Univariate risk evaluation for permanent colonization during the whole length of stay at the ICU (D1-D8) *.

Variable	Categories	Colonized n (%)	Odds Ratio (95% CI)	*p*
ICU Unit	BAH	17/60 (28.3)	1	-
	Chirurgical FFH	5/16 (31.3)	1.15 (0.35-3.81)	0.819
	General FFH	4/13 (30.8)	1.12 (0.30-4.14)	0.860
Age range (years)	18-40	2/7 (28.6)	1	-
	41-60	7/21 (33.3)	1.25 (0.19-8.14)	0.815
	61-80	15/54 (27.8)	1.11 (018-6.99)	0.911
	81+	2/7 (28.6)	1.00 (0.10-10.17)	1.000
Gender	Female	9/34 (26.5)	1	0.655
	Male	17/55 (30.9)	1.24 (0.48-3.22)	-
Race	Melanodermic	6/14 (42.9)	1	-
	Leucodermic	20/75 (26.7)	0.48 (0.15-1.57)	0.228
Diabetes mellitus	No	14/56 (25.0)	1	-
	Yes	12/33 (36.4)	1.71 (0.67-4.35)	0.255
Pulmonary infection	No	16/54 (29.6)	1	-
	Yes	10/35 (28.6)	0.95 (0.37-2.43)	0.915

Cardiovascular disease	No	26/80 (32.5)	1	-
	Yes	0/9 (0.0)	-	NA
Solid tumor	No	18/69 (26.1)	1	-
	Yes	8/20 (40.0)	1.89 (0.66-5.36)	0.228
Gastrointestinal pathology	No	19/68 (27.9)	1	-
	Yes	7/21 (33.3)	1.29 (0.45-3.69)	0.635
Anemia (Hb < 10 mg/dL)	No	22/81 (27.2)	1	-
	Yes	4/8 (50.0)	2.68 (0.62-11.66)	0.188
Urinary tract infection	No	22/83 (26.5)	1	-
	Yes	4/6 (66.7)	5.54 (0.95-32.43)	0.057
COVID-19	No	24/85 (28.2)	1	-
	Yes	2/4 (50.0)	2.54 (0.34-19.08)	0.364
HIV/AIDS	No	25/87 (28.7)	1	-
	Yes	1/2 (50.0)	2.48 (0.15-41.21)	0.526
Hematological malignancy	No	24/86 (27.9)	1	-
	Yes	2/3 (66.7)	5.17 (0.45-59.65)	0.188
Severe immunodeficiency	No	25/87 (28.7)	1	-
	Yes	1/2 (50.0)	2.48 (0.15-41.21)	0.526
Vesical catheter	No	8/27 (29.6)	1	-
	Yes	18/62 (29.0)	0.97 (0.36-2.62)	0.955
Presence of CVC	No	8/29 (27.6)	1	-
	Yes	18/60 (30.0)	1.12 (0.42-3.01)	0.814
Mechanical ventilation	No	12/48 (25.0)	1	-
	Yes	14/41 (34.1)	1.56 (0.62-3.90)	0.344
Abd. surgery	No	23/74 (31.1)	1	-
	Yes	3/15 (20.0)	0.55 (0.14-2.15)	0.394
Dialysis	No	23/82 (28.0)	1	-
	Yes	3/7 (42.9)	1.92 (0.40-9.27)	0.415
Total parental nutrition	No	26/89 (29.2)	1	-
	Yes	0/0 (0.0)	-	NA
Neutropenia	No	26/88 (29.5)	1	-
	Yes	0/1 (0.0)	-	NA
Antibiotic therapy	No	6/28 (21.4)	1	-
	Yes	20/61 (32.8)	1.79 (0.63-5.11)	0.274
Treatment with antifungal agents	No	24/83 (28.9)	1	-
	Yes	2/6 (33.3)	1.23 (0.21-7.16)	0.819

* 26 (29.2%) out of 89 patients that remained during the whole length of stay at the ICU were permanently colonized.
